# Draft Genome Sequence of *Thalassotalea* sp. Strain ND16A Isolated from Eastern Mediterranean Sea Water Collected from a Depth of 1,055 Meters

**DOI:** 10.1128/genomeA.01231-14

**Published:** 2014-11-26

**Authors:** Savannah C. Stelling, Stephen M. Techtmann, Sagar M. Utturkar, Noor K. Alshibli, Steven D. Brown, Terry C. Hazen

**Affiliations:** a Department of Civil and Environmental Engineering, University of Tennessee, Knoxville, Tennessee, USA; b Center for Environmental Biotechnology, University of Tennessee, Knoxville, Tennessee, USA; c Biosciences Division, Oak Ridge National Laboratory, Oak Ridge, Tennessee, USA; d Graduate School of Genome Science and Technology, University of Tennessee, Knoxville, Tennessee, USA

## Abstract

*Thalassotalea* sp. strain ND16A belongs to the family *Colwelliaceae* and was isolated from eastern Mediterranean Sea water at a depth of 1,055 m. Members of *Colwelliaceae* are ubiquitous marine heterotrophs. Here, we report the draft genome sequence of *Thalassotalea* sp. strain ND16A, a member of the newly described genus *Thalassotalea*.

## GENOME ANNOUNCEMENT

The gammaproteobacterial family *Colwelliaceae* contains the genera *Colwellia* and *Thalassamonas*, as well as the newly described genus *Thalassotalea* (1). These bacteria are found in the coastal and deep ocean and in sea ice (1, 2). The *Colwelliaceae* are aerobic chemo-organoheterotrophic microbes (1, 2). Members of this family have a very broad range of growth temperatures, with many of the *Colwellia* and *Thalassotalea* species able to grow at 4°C. Recently, members of the *Colwelliaceae* were shown to degrade hydrocarbons as important members of the microbial community that responded to the Deepwater Horizon oil spill (3–8). The first sequenced member of the *Colwelliaceae* was *Colwellia psychrerythraea* 34H (2). Currently, four *Colwellia* genomes are publicly available (*Colwellia psychrerythraea* 34H [GenBank accession number CP000083.1], *Colwellia piezophila* [GenBank accession number ARKQ00000000.1], *Colwellia* sp. 8_GOM-1096m [GenBank accession number JONX00000000.1], and *Colwellia* sp. MT41 [BioProject IMG number 250165125]). Minimal information is available about other genera in the *Colwelliaceae* due to unavailability of sequenced genomes.

*Thalassotalea* sp. strain ND16A was isolated from eastern Mediterranean Sea water at a depth of 1,055 m and a temperature of 13.8°C (29.821°E, 31.804°N). Strain ND16A was grown at 14°C on ONR7a medium (9) supplemented with 100 ppm of regional oil (Norne Blend) as the carbon source.

The draft genome sequence for *Thalassotalea* sp. strain ND16A was generated using the Illumina MiSeq platform, which generated 1,462,477 paired-end reads. Quality-based trimming was performed using Trimmomatic with the following parameters: SLIDINGWINDOW:4:15 MINLEN:36 (10). After quality filtering, 1,294,324 paired-end reads remained, resulting in 430,552,2922 bp of sequence data with an average read length of 266 bp. After testing several approaches (11), the genome was assembled using SPAdes version 3.1 (12) into 130 large (≥500 bp) contigs, with a total genome size of 4.6 Mb. The *N*_50_ contig size was 108,374 bp, with the largest contig being 336,715 bp. Genes were predicted using the Prodigal algorithm (13) as part of the Oak Ridge National Laboratory genome annotation pipeline.

The draft genome has an overall G+C content of 42.2% and 3,953 candidate protein-encoding genes. Putative functions from COG functional groups were assigned to 76% of the candidate genes. RNAMMer (14) predicted eight 5S rRNA, one 16S rRNA, and one 23S rRNA gene. Additionally, tRNAscan-SE (15) identified 74 tRNAs, with tRNAs for each of the 20 amino acids. Our analysis, based on 16S rRNA gene phylogeny, showed that ND16A is monophyletic with other *Thalassotalea* species and clearly separates from the *Thalassomonas* and *Colwellia* groups. The genome of strain ND16A contains 39 oxygenase genes, including a chatechol 2,3 dioxygenase and two ring-hydroxylating dioxygenase gene clusters, which are important in aromatic hydrocarbon degradation. These findings in part explain the ability of ND16A to grow on oil. The genome of strain ND16A provides the first genome of a member of the recently described genus *Thallasolatea* and may shed light on the oil-degrading ability of members of the *Colwelliaceae*.

### Nucleotide sequence accession number.

The draft genome sequence of strain ND16A has been deposited at DDBJ/EMBL/GenBank under the accession number JQDZ00000000. The version described in this paper is the first version.
